# Crystal Structure of Mouse Thymidylate Synthase in Tertiary Complex with dUMP and Raltitrexed Reveals N-Terminus Architecture and Two Different Active Site Conformations

**DOI:** 10.1155/2014/945803

**Published:** 2014-06-03

**Authors:** Anna Dowierciał, Piotr Wilk, Wojciech Rypniewski, Wojciech Rode, Adam Jarmuła

**Affiliations:** ^1^Nencki Institute of Experimental Biology, Polish Academy of Sciences, 3 Pasteur Street, 02-093 Warsaw, Poland; ^2^Institute of Bioorganic Chemistry, Polish Academy of Sciences, 12/14 Noskowskiego Street, 61-704 Poznań, Poland

## Abstract

The crystal structure of mouse thymidylate synthase (mTS) in complex with substrate dUMP and antifolate inhibitor Raltitrexed is reported. The structure reveals, for the first time in the group of mammalian TS structures, a well-ordered segment of 13 N-terminal amino acids, whose ordered conformation is stabilized due to specific crystal packing. The structure consists of two homodimers, differing in conformation, one being more closed (dimer AB) and thus supporting tighter binding of ligands, and the other being more open (dimer CD) and thus allowing weaker binding of ligands. This difference indicates an asymmetrical effect of the binding of Raltitrexed to two independent mTS molecules. Conformational changes leading to a ligand-induced closing of the active site cleft are observed by comparing the crystal structures of mTS in three different states along the catalytic pathway: ligand-free, dUMP-bound, and dUMP- and Raltitrexed-bound. Possible interaction routes between hydrophobic residues of the mTS protein N-terminal segment and the active site are also discussed.

## 1. Introduction

Thymidylate synthase (TS; EC 2.1.1.45), a target enzyme in antitumor chemotherapy [1], catalyzes the N(5,10)-methylenetetrahydrofolate- (mTHF-) dependent C(5)-methylation of deoxyuridylate (dUMP), leading to the formation of thymidylate (dTMP), a nucleotide required for DNA biosynthesis [2]. The enzyme, being a homodimer, is strongly conserved among the prokaryotic and eukaryotic species, from bacteria to mammals. A feature distinguishing mammalian from bacterial TS enzymes is N-terminal extension by some 27 amino acid residues, present in the former but absent in the latter enzymes. However, that protein segment is usually disordered and thus absent in the crystal structures of a mammalian TS [3]. The present paper reports the first structure of mammalian TS—the tertiary complex of mouse TS (mTS) with dUMP and the strong antifolate inhibitor and anticancer drug, Raltitrexed—with a clearly defined segment of 13 N-terminal amino acids.

Several crystal structures of mammalian (human, rat, or mouse) TSs have been thus far studied, revealing a range of states along the catalytic pathway, from unliganded, inactive through liganded, active and open to liganded, active and closed. Based on the crystal structures of rat TS (rTS) in complex with dUMP and Raltitrexed [4] and an analogous complex of a deletion mutant of human TS (hTS) [5], Raltitrexed has been postulated to competitively inhibit TS by preventing formation of a covalent bond between the enzyme catalytic cysteine and the dUMP pyrimidine ring. However, in another structure, representing the full-sequence hTS protein in a complex with dUMP and Raltitrexed, the closed enzyme conformation and covalently bound substrate were observed [6]. The present structure of the mTS-dUMP-Raltitrexed complex shows an interesting mixture of open and closed conformations, suggesting an asymmetrical influence of Raltitrexed binding on the subunit conformations in the two crystallographically independent protein molecules.

## 2. Materials and Methods

### 2.1. Protein Preparation and Crystallization

Recombinant mouse TS protein, overexpressed and purified as described previously [7], was dialyzed against 5 mM Tris HCl buffer, containing 5 mM DTT and cocrystallized with the ligands at 4°C in the hanging drop composed of equal volumes (3.2 *μ*L each) of the protein solution (0.9 mM mTS), containing 6 mM dUMP and 6 mM Raltitrexed, and well solutions, containing 0.1 M MES pH 5.5–5.8, 5 mM DTT, 15.5% PEG 5K MME, and 0.2 M (NH_4_)_2_SO_4_.

### 2.2. Crystal Diffraction and Structure Determination

X-ray diffraction data were collected from a single flash-frozen crystal at the BESSY Synchrotron in Berlin, Germany, using X-rays of a 0.918 Å wavelength. Data were processed with* Denzo* and* Scalepack* [8]. The space group was P2_1_2_1_2_1_. The structure was solved by molecular replacement using the structure of mouse thymidylate synthase complexed with an inhibitor N(4)-OH-dCMP as a search model and the program* Phaser* [9] from the CCP4 package [10] to yield an asymmetric unit comprising two homodimers of the mTS enzyme, containing in all four active sites (two per each dimer) single molecules of dUMP and Raltitrexed. Refinement and model building was carried out using* Refmac5* [11] from CCP4 and* Coot* [12]. Water molecules were added automatically using* Refmac5* and picked manually. Model validation was performed using* Sfcheck* [13] and* Procheck* [14] in CCP4. Data collection and refinement statistics are given in Table 1. Figures were prepared with* Coot* or* VMD* [15] and rendered with* Raster3D* [16].

## 3. Results and Discussion

**Figure 1:**
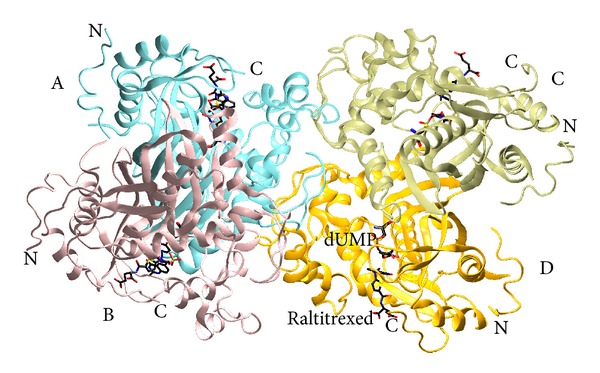
Two homodimers of the mTS-dUMP-Raltitrexed complex structure, AB and CD, in the asymmetric unit. Subunits A, B, C, and D are colored differently and labeled. The ligands, dUMP and Raltitrexed, are colored according to atom identity (carbon in black, oxygen in red, nitrogen in blue, and sulfur and phosphorus in yellow), shown as sticks, and labeled in subunit D. N- and C-termini are labeled in all subunits. The enzyme belongs to the class of *α* and *β* proteins (*α* + *β*), with the dimer subunits separated by two reversely symmetrical large mixed *β*-sheets.

The crystal structure of mouse thymidylate synthase (mTS) in complex with dUMP and Raltitrexed comprises two homodimers, AB and CD, consisting of chains A, B, C, and D (Figure 1), represented by continuous and (overall) well-defined electron densities for amino acids 21–307, 20–307, 21–302, and 21–304, respectively. The electron density is overall slightly better defined for the dimer AB than CD (not shown). The ligands, dUMP and Raltitrexed, are described by clear electron densities in all subunits. Unexpectedly, an N-terminal segment consisting of residues 1–13, usually disordered in mammalian thymidylate synthases, is clearly visible in all subunits, even though the following segment 14–20 (14–19 in chain B) is not visible on the electron density maps and has not been included in the final model.

**Figure 2:**
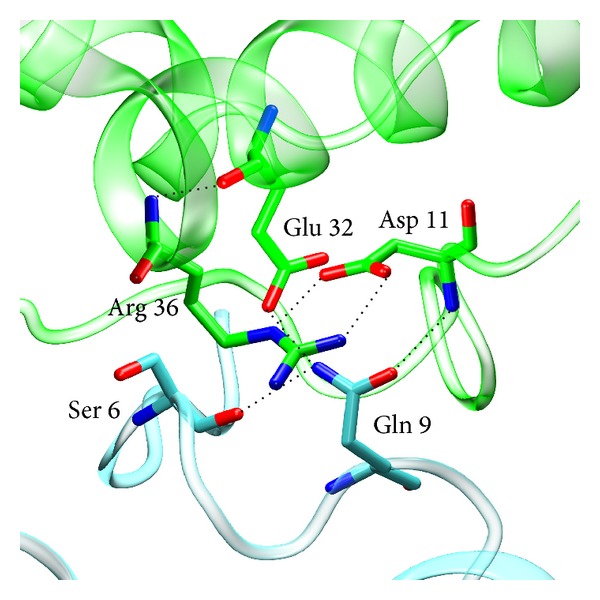
Hydrogen bonding network between Gln9 and Ser6 from a given molecule of the mTS-dUMP-Raltitrexed complex structure (blue) and Glu32 and Asp11 from a neighboring molecule in the crystal lattice (green).

Of note is that Ramachandran's plot analysis indicates unfavored conformations of Gln9 from all subunits. The unusual conformations of Gln9 appear to be stabilized by intermolecular hydrogen bonding network connecting the side chain groups of Gln9 and Ser6 with Asp11 and Glu32 from a neighboring molecule in the crystal lattice (Figure 2).

### 3.1. Homodimers AB and CD

Comparison of the liganded subunits shows that the chains A and B in the dimer AB are overall more similar to each other than the chains C and D in the dimer CD (reflected by the C*α* RMSD values of 0.384 Å between the whole chains A and B and 0.642 Å between the whole chains C and D, as well as 0.387 Å between the protein cores (alone, with the N-terminal residues 1–13 omitted) A and B and 0.618 Å between the protein cores C and D). The subunit active sites are similar within each dimer but significantly different between the two dimers AB and CD, with the latter being related to the degree of the closure of a given active site.

**Figure 3:**
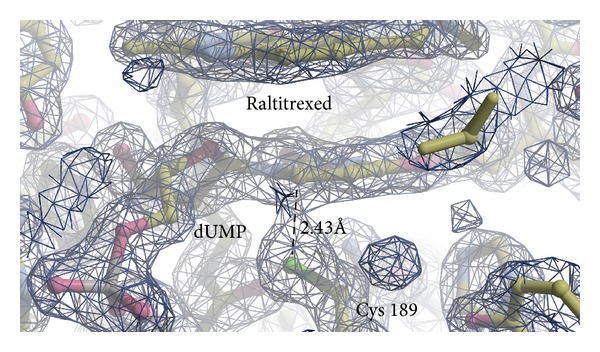
2Fo-Fc electron density map at 1.8σ level showing dUMP, Raltitrexed, and catalytic Cys189 in the subunit B. Continuous electron density can be seen between Cys189 *γ*S and dUMP C6 atoms. The *γ*S-C6 distance of 2.43 Å is marked by dashed line.

The comparison of both dimers with previously described analogous complexes of* E. coli* TS (*Ec*TS, PDB ID: 2KCE) [17], populating the closed conformation,* C. elegans* TS (*Ce*TS, PDB ID: 4IQQ; unpublished), populating the open conformation, rat TS (rTS, PDB ID: 2TSR) [4], populating the open conformation and two complexes of human TS, one populating the closed conformation (hTS, PDB ID: 1HVY) [6] and the other one the open conformation (hTS, PDB ID: 1I00) [5], indicates a mixed (partly closed and partly open) character of the active site conformations observed in both dimers of the mouse enzyme. However, the “contributions” of the closed and open conformations are different in the two dimers, with the dimer AB resembling more closely the closed conformation seen in the 1HVY structure (with the C*α* RMSD values for mTS-dUMP-Raltitrexed (chain A, residues 21–307) versus 1HVY (chain A, residues 27–313) and versus 2TSR (chain A, residues 21–301) being 0.368 Å and 0.750 Å, resp.) and dimer CD resembling closer the open conformation seen in the 2TSR structure (with C*α* RMSD for mTS-dUMP-Raltitrexed (chain C, residues 21–307) versus 1HVY (chain A, residues 27–313) and versus 2TSR (chain A, residues 21–301) being 0.857 Å and 0.474 Å, resp.). The latter is in accord with the corresponding distances between mTS Cys189 *γ*S and dUMP C6 that, albeit in all cases too long to indicate covalent bonds, are shorter and thus indicative of a tighter binding of ligands in more closed dimer AB (2.48 and 2.43 Å for chains A and B, resp.) than in more open dimer CD (2.78 and 2.76 Å for chains C and D, resp.). Moreover, considering noticeable electron densities between mTS Cys189 *γ*S and dUMP C6 in the subunits A and B, including continuous electron density at 1.8σ in the latter subunit (Figure 3), the presence of the *γ*S-C6 covalent bond in some fraction of the population of mTS-dUMP-Raltitrexed molecules may be expected. This is further reflected in the above mentioned *γ*S-C6 bond distances in the AB dimer and, to a lesser extent, the CD dimer, which are distinctly shorter than the sum of van der Waals radii (3.5 Å) for the sulfur (1.8 Å) and carbon (1.7 Å) atoms. In addition, in accord with more closed conformation of the dimer AB than CD, the C-terminal segment that contributes to the active site closing and enzyme catalysis is described by a well-defined electron density throughout the segment in the former, in contrast to a weaker electron density, resulting in the lack of a few most terminal residues in the latter. Furthermore, the analysis of solvent accessible surface area (SASA) shows the dimer AB to be more compact, that is, more closed, compared to the dimer CD (SASA for chains A, B, C, and D is 10121.2, 10814.9, 11099.1, and 10952.4 Å^2^, resp.). Altogether, these results show Raltitrexed binding to influence the present structure in an asymmetric manner, changing the conformation of one protein dimer (AB) more than the other (CD). The cause of this asymmetry in a single structure is not quite clear but there are some differences in the crystal packing that could help in stabilizing the two different conformations. Those differences can be observed in the crystal lattice neighborhoods of chains A and B relative to chains C and D (not shown); for the two former chains, in the vicinity of an active site cleft there is contiguous electron density, symmetrical to dimer CD, and additional H-bonds are possible, that is, between Glu42 (chains A and B) and Asn296 (chains symmetrical to D and C) and between Gly46 (chains A and B) and Arg 268 (chains symmetrical to D and C). These H-bonds are not possible for chains C and D and therefore may help to stabilize the more closed conformation of dimer AB compared to CD.

**Figure 4:**
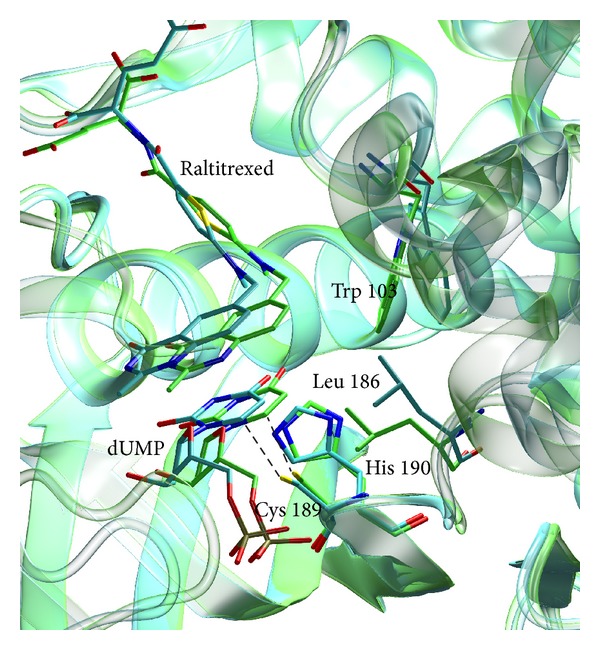
Superimposition of subunits A (green) and C (blue) of the mTS-dUMP-Raltitrexed complex structure. Ligands and selected active site residues, which differ conformationally between subunits A and C, are shown as sticks. The Cys189 *γ*S-dUMP C6 distances of 2.48 Å (subunit A) and 2.78 Å (subunit C) are marked by dashed lines.

A detailed comparison of the subunits in the mTS-dUMP-Raltitrexed structure shows differences in the orientation of the active site residues Trp103 and Leu186 between the more closed subunit A and the more open subunit C (Figure 4). It has been shown that in* E. coli *TS Trp80 (equivalent to mouse Trp103) is responsible for the proper orientations of Leu143 (equivalent to mouse Leu186) and of the cofactor molecule and that in the closed conformation the side chain of Leu143 plays a role in sequestering the active site from solvent [18]. Such a barrier for water molecules, formed by Leu186, is observed in chain A, but not in chain C, with orientations of Leu186 and Trp103 in the latter resembling those found in the previously presented binary mTS-dUMP complex [7].

### 3.2. The N-Terminus

**Figure 5:**
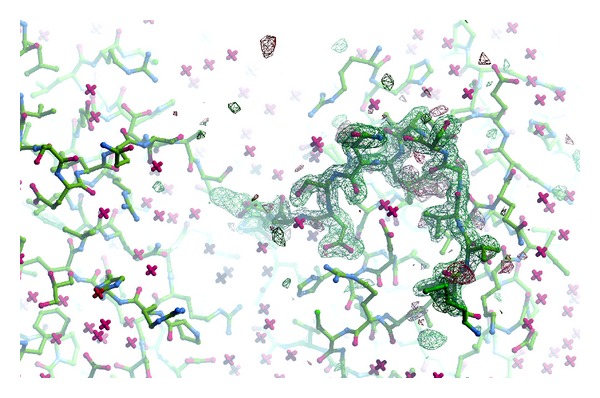
Omit difference map for N-terminal residues 1–13 in subunit A, contoured at 3σ.

In mammalian TSs, the N-terminus, 25–29 residues longer than in bacterial enzyme forms and suggested to play an important role in protein turnover [19, 20], is usually not visible in crystal structures. So far three structures of mutated human TSs (PDB IDs: 3ED7, 3GH0, and 3GH2) allowed to view together only a few N-terminal amino acids [3]. Surprisingly, in the course of the mTS-dUMP-Raltitrexed complex structure refinement a large unmodeled segment of a clear electron density was observed at the N-terminal region in each subunit. This was interpreted as a 13-residue segment (Met1-Gln13) (Figure 5). The segment was stable enough to show up unambiguously on the electron density maps, probably owing to crystal packing, rather than intramolecular interactions. Supplementary Figure 1S available online at http://dx.doi.org/10.1155/2014/945803, shows two antiparallel arrays of several nonpolar residues from two neighboring dimers (Met1, Leu2, Val3, Val4, Gly5, and Leu8) that stabilize the crystal packing by exerting strong hydrophobic interactions between each other. It should be stressed, though, that while the conformation of the Met1-Gln13 segment is stabilized by intermolecular contacts, its structure can be reasonably assumed to represent some energy minimum and a meaningful instance from a range of possible conformations.

**Figure 6:**
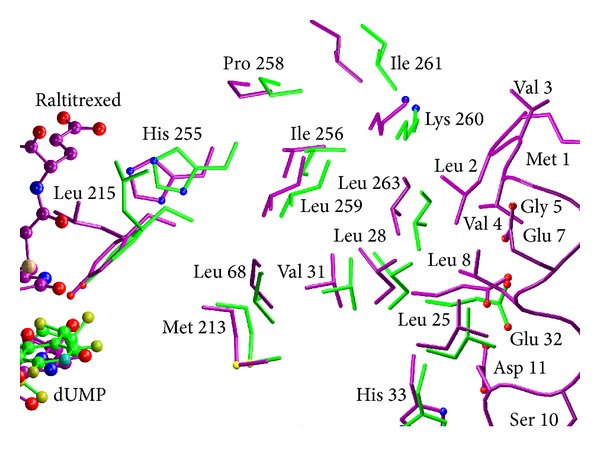
Superimposition of the crystal structures of mTS-dUMP (green) and mTS-dUMP-Raltitrexed (violet) complexes. Side chains of selected, mostly hydrophobic, amino acid residues located between the N-terminus and active site are shown as sticks and ligands, dUMP and Raltitrexed, as ball and sticks. N-terminal amino acids in the tertiary complex have no equivalents in the binary complex, as this part was disordered in the latter structure.

**Figure 7:**
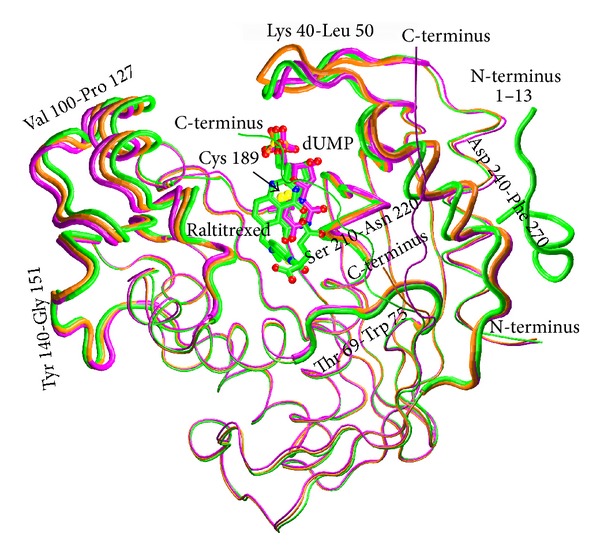
Superimposition of the main chains of the three structures: ligand-free mTS (orange, PDB ID: 3IHI), mTS-dUMP complex (violet, PDB ID: 4E5O), and mTS-dUMP-Raltitrexed complex (green, PDB ID: 4EB4). Ligands, dUMP and Raltitrexed, and catalytic Cys189 are shown as sticks. N, O, and S atoms are colored blue, red, and yellow, respectively. C atoms are colored the same as the corresponding tubes representing the main chains of respective complexes. The most differing main chain fragments are shown as thicker tubes. The 13-residue N-terminal fragment (1–13) in the tertiary complex, having no equivalents in the ligand-free enzyme and the binary complex, is also shown.

Mutations in the N-terminus were recently shown to affect the enzyme's catalytic activity [3]. It was observed that V3F mutation in hTS significantly lowered enzyme activity, and the crystal structure of this mutated construct in complex with FdUMP revealed “wrong” binding location of the ligand molecule as a probable cause. In another mutant, V3L, a sharp drop in the catalytic activity has been assigned to Leu3 acting as a stabilizer of the inactive conformation of loop 181–197, with the latter preventing substrate binding. Comparison of the mTS binary (PDB ID 4E5O) [7] and tertiary complex presented here points to possible influence of nonpolar residues in the N-terminus on residues located in the active site through a series of hydrophobic interactions, including those between Leu2 and Val4, and amino acids in the protein interior, in particular Ile261 and Leu263 (Figure 6). The latter two and other residues located in the protein segment 240–270, with the latter undergoing a considerable shift upon binding of Raltitrexed (Figure 7), may in turn interact with amino acids located in the proximity of the active site. Ultimately, the most notable effect of such interaction routes would be a change in the position of the side chain of Leu215, a residue located in the direct vicinity of Raltitrexed (Figure 6). Considering the relatively large sequence differences in the TS N-terminal region between various species, it may be a good idea to explore this region in future studies as a target for species-specific, allosteric interactions with the enzyme active site.

### 3.3. Comparison of Ligand-Free, dUMP-Bound and dUMP- and Raltitrexed-Bound Structures of Mouse TS

Conformational changes resulting in a ligand-induced closure of the active site cleft can be tracked by comparing the crystal structures of mouse thymidylate synthase in three distinct states along the catalytic pathway: ligand-free [7], dUMP-bound [7], and dUMP- and Raltitrexed-bound. Figure 7 shows the latter three structures superimposed with the major differences emphasized. Most significant differences are apparent in two loops flanking the entrance to the active site cleft, 100–127 and 40–50, another loop surrounding the cleft, 240–270, and the C-terminus. The binding of dUMP has an effect on the loop 100–127, causing it to move away from the center of the active site entrance and thus widening the latter. The effect is reversed upon the binding of Raltitrexed, when with the two ligands being already in place the loop 100–127 moves back toward the center of the active site entrance and partly seals the latter. Placed as in the tertiary complex, the conformation of the loop 100–127 is consolidated by favorable contacts of Ile102, Trp103, and Asn106 with Raltitrexed. The loop 40–50, located on the opposite flank of the active site entrance with respect to the loop 100–127, reacts to the binding of dUMP by shifting toward the center of the active site, causing the formation of a stabilizing hydrogen bond between Arg44 and the phosphate moiety of dUMP. Unlike with the loop 100–127, though, the position of the loop 40–50 alters only slightly in the tertiary complex. The third loop that reacts to the binding of ligands by a shift leading to tightening of the active site cleft encompasses amino acid residues 240–270. It shifts slightly upon binding of dUMP in the binary complex, but much more substantially following Raltitrexed binding in the tertiary complex. Its conformation is stabilized by hydrogen bonding between the invariant His250 and Tyr252 from the loop and the hydroxyl group of the deoxyribose moiety in dUMP (the latter being the primary contacts of the deoxyribose ring of dUMP [21]) and additionally, only in the tertiary complex, by a hydrophobic contact between the phenyl ring of Tyr252 and the quinazoline ring of Raltitrexed. Finally, in the dUMP- and Raltitrexed-bound structure, the C-terminal region is found to follow the active site conformational change and partly cover its entrance, whereas in the ligand-free and dUMP-bound structures the courses of the latter region are very different and leave the entrance uncovered. Oriented as in the tertiary complex, the conformation of the C-terminus is stabilized by hydrophobic contacts between Lys302, Met305, and Ala306 and Raltitrexed. It should be noted that none of the reported differences affect the interface region between the subunits of the mTS dimer, being very similar in the ligand-free and both ligand-bound structures (not shown).

Comparison of the active site clefts indicates differences in the conformation of a few residues involved in the binding of ligands, including Tyr252 and Arg44, whose conformational changes coupled to their ligand-binding roles have been described in the preceding paragraph, and Trp103 that reorients to form one of the key hydrophobic contacts stabilizing the position of Raltitrexed in the tertiary complex.

## 4. Conclusions

The crystal structure of the tertiary complex between the mouse thymidylate synthase enzyme and the ligands, dUMP and Raltitrexed, shows an asymmetrical influence of the binding of the latter ligand on the conformation of two crystallographically independent complex molecules. The more closed molecule of the complex dimer AB allows a tighter binding of ligands, reflected in both closer distances and increased electron densities between dUMP pyrimidine ring C6 atom and enzyme catalytic Cys189 *γ*S atom in this molecule, suggestive of the presence of the C6-*γ*S covalent bond in a fraction of mTS-dUMP-Raltitrexed molecules. The present structure reveals the architecture of a 13-residue N-terminal segment, visible for the first time among the crystal structures of mammalian TSs. This segment seems to be able to communicate with the enzyme active site through a series of hydrophobic interactions. Considering relative diversity of the N-terminal region among TSs from various organisms, such allosteric-like effects may be worth further exploration in view of possible implications for species-specific drug design.

## Supplementary Material

Legend to Supplementary Figure 1S: Two antiparallel arrays of N-terminal non-polar residues stabilizing the molecular crystal packing of the mTS-dUMP-Raltitrexed complex structure.

## Figures and Tables

**Table 1:** Data collection and refinement statistics/PDB code 4EB4. Values in parentheses are for the highest resolution shell.

Data collection	
Space group	P2_1_2_1_2_1_
Cell dimensions	*a* = 101.80 Å, *b* = 114.22 Å, *c* = 123.65 Å *α* = *β* = *γ* = 90°
Beamline	Bessy 14.1
Wavelength [Å]	0.918
Resolution [Å]	20.04–1.74 (1.76–1.74)
*R*_merge_*	0.054 (0.556)
Unique reflections	148229
Completeness [%]	99.9 (100)
Redundancy	3.6 (3.5)
〈*I*/*σ*(*I*)〉	21

Refinement	
Resolution [Å]	1.74 (1.78–1.74)
Number of reflections	148148 (9725)
R**	0.19 (0.31)
*R*_free_***	0.24 (37)
Average B factor [Å^2^]:	
Overall	31
Polypeptide chain A	24
Polypeptide chain B	24
Polypeptide chain C	35
Polypeptide chain D	36

RMS deviations from ideal values for refined atoms	
Bond lengths [Å]	0.022
Bond angles [°]	1.967

Ramachandran's plot assignments	
The most favored	936 (92.0%)
Additionally allowed	77 (7.6%)
Generously allowed	4 (0.4%)
Disallowed	0 (0.0%)

*R_merge_ = ∑_hkl_∑_*i*_|*I*_*i*_(hkl) − 〈*I*(hkl)〉|/∑_hkl_∑_*i*_ *I*_*i*_(hkl), where *I*_*i*_(hkl) is the integrated intensity of a given reflection and 〈*I*(hkl)〉 is the mean intensity of multiple corresponding symmetry-related reflections.

**R = ∑_hkl_||*F*_obs_| − |*F*_calc_||/∑_hkl_|*F*_obs_|, where *F*_obs_ and *F*_calc_ are the observed and calculated structure factors, respectively.

***R_free_ is *R* calculated using a randomly chosen 1000 reflections that were excluded from the refinement.

## Additional Data

The atomic coordinates and structure factors have been deposited in Protein Data Bank under accession code 4EB4.
