# Supplementary material for: Crystal Structure of Mouse Thymidylate Synthase in Tertiary Complex with dUMP and Raltitrexed Reveals N-Terminus Architecture and Two Different Active Site Conformations
Source: Biomed Res Int. 2014 Jun 3;2014:945803. doi: 10.1155/2014/945803 (PMC4065713; doi:10.1155/2014/945803)
Supplement: Supplementary file 1 — Legend to Supplementary Figure 1S: Two antiparallel arrays of N-terminal non-polar residues stabilizing the molecular crystal packing of the mTS-dUMP-Raltitrexed complex structure. [file 945803.f1.pdf]

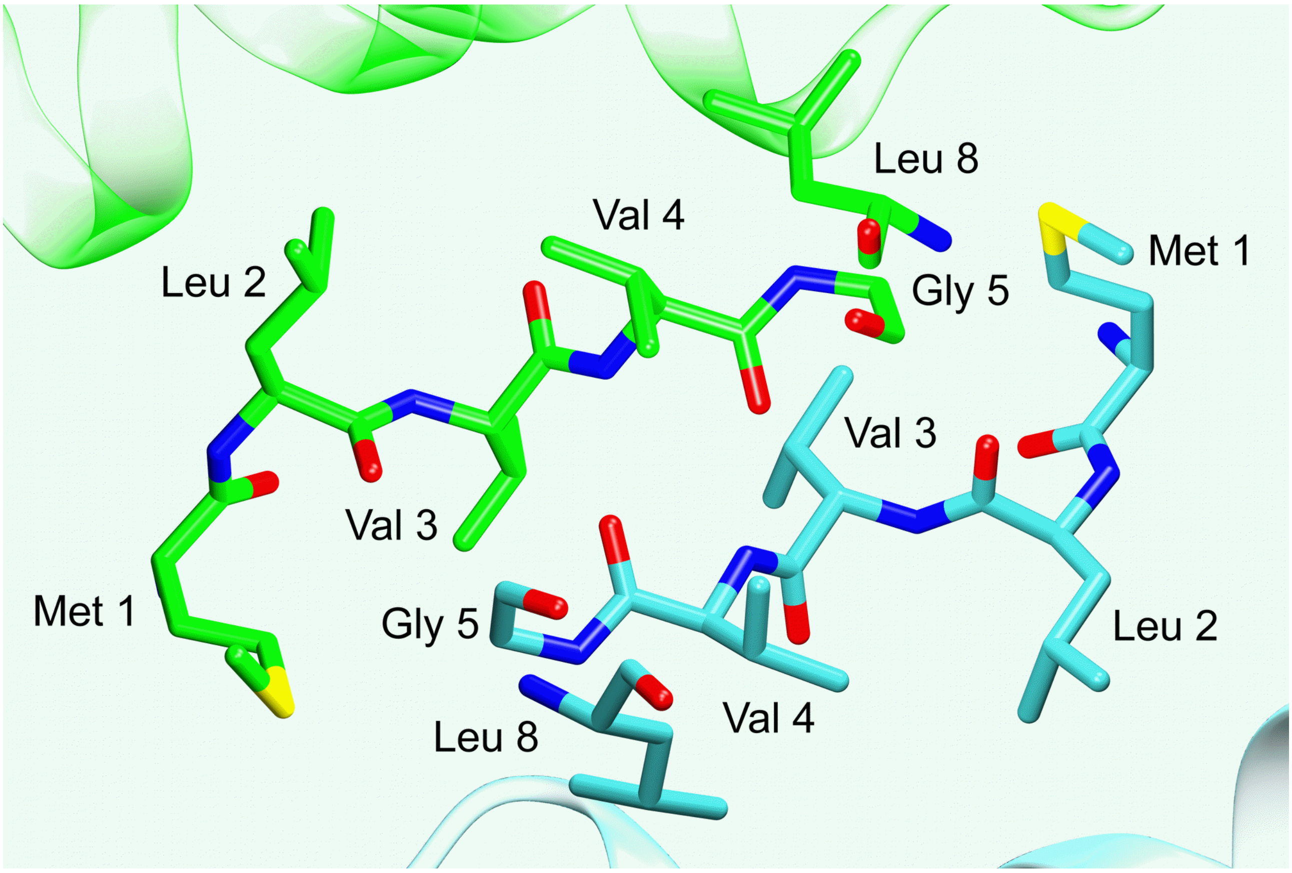

Fig. 1S. Two antiparallel arrays of N-terminal non-polar residues stabilizing the molecular crystal packing of the mTS-dUMP-Raltitrexed complex structure.
